# NXN suppresses metastasis of hepatocellular carcinoma by promoting degradation of Snail through binding to DUB3

**DOI:** 10.1038/s41419-022-05135-7

**Published:** 2022-08-04

**Authors:** Yuanping Zhang, Dinglan Zuo, Jiliang Qiu, Kai Li, Yi Niu, Yichuan Yuan, Yuxiong Qiu, Liang Qiao, Wei He, Chenwei Wang, Yunfei Yuan, Binkui Li

**Affiliations:** 1 State Key Laboratory of Oncology in South China and Collaborative Innovation Center for Cancer Medicine, Sun Yat-Sen University Cancer Center, Guangzhou, China; 2 Department of Liver Surgery, Sun Yat-Sen University Cancer Center, Guangzhou, China

**Keywords:** Tumour-suppressor proteins, Metastasis

## Abstract

The poor prognosis of hepatocellular carcinoma (HCC) could be attributed to its high metastasis rate. Here, we report the role of nucleoredoxin (NXN), a multifunctional redox-active protein, in HCC metastasis. The expression of NXN in HCC tissues was measured by immunohistochemistry. The role of NXN on HCC proliferation was determined by CCK-8, EdU and colony formation assays in vitro and subcutaneous tumor formation model in vivo. Transwell and wound healing assays and tail vein injection model were performed to assess the function of NXN on HCC metastasis. Co-immunoprecipitation assay was performed to examine the interaction among NXN, Snail and DUB3. Our results showed that NXN was downregulated in HCC tissues compared to adjacent liver tissues. Patients with low NXN expression had shorter overall survival (OS) time (*P* < 0.001) than those with high NXN expression. Biologically, ectopic expression of NXN significantly inhibited the proliferation and metastasis of HCC cells both in vitro and in vivo by suppressing epithelial-mesenchymal transition (EMT). Mechanistically, NXN promoted the ubiquitin-proteasome-mediated degradation of Snail through interaction with DUB3. Further, depletion of Snail abolished NXN-inhibited cell proliferation and metastasis. In summary, NXN suppressed the proliferation and metastasis of HCC by inhibiting DUB3-mediated deubiquitylation of Snail protein. Our study demonstrates that NXN, DUB3 and Snail complex functioned as an important regulatory mechanism of HCC progression and indicates a potential therapeutic approach for the treatment of HCC metastasis.

## Introduction

Hepatocellular carcinoma (HCC) is one of the most common malignancies worldwide [1, 2], and the high incidence of its recurrence or metastasis contributes to the poor prognosis of HCC patients [3, 4]. Therefore, explorations of the underlying molecular mechanisms of HCC metastasis are urgently needed to develop novel therapeutic strategies and prolong the patients’ survival.

The epithelial-mesenchymal transition (EMT) has important roles in all stages of cancer progression from initiation, invasion and metastasis to colonization [5–7]. EMT is executed by EMT‑activating transcription factors (EMT‑TFs), consisting mainly of the Snail, Twist and ZEB families [8]. Snail (encoded by *SNAI1*) is a key transcriptional repressor of E-cadherin expression that can regulate tumor metastasis [8]. Many studies have found that the process of ubiquitination and de-ubiquitination plays a critical role in maintaining the stability and homeostasis of Snail. By hydrolyzing ubiquitin from the protein substrate, DUB3, USP29 and USP1 were found to stabilize the Snail protein [9–11].

We previously found that the methylation signature of genes could predict recurrence in HCC patients [12]. Among them, Nucleoredoxin (NXN, cg19987768) was highly methylated in early-stage HCC patients with recurrence compared to those patients without recurrence, which prompted that NXN might be correlated with recurrence in HCC. NXN, also known as TRG-4, NRX or RRS2, is a member of the thioredoxin (TRX) family that is related to cell growth and differentiation [13, 14]. It has been reported that NXN played a role in liver fibrosis and polycystic liver disease [15–17]. In breast cancer, NXN participated in retinoic acid-mediated apoptosis [18]. Whereas, little is known about the expression and role of NXN in HCC.

In this present study, we found that NXN was downregulated in HCC, and its low expression correlated with poor overall survival (OS) in HCC patients. By inhibiting the EMT process, NXN overexpression was found to suppress the proliferation ability and metastatic potential of HCC cells both in vitro and in vivo. By interacting with DUB3, NXN could reduce the interaction ratio of Snail and DUB3 and subsequently promote the ubiquitin-proteasome-mediated degradation of the Snail protein. These findings highlight that NXN played a critical role in HCC metastasis, suggesting NXN as a potential biomarker and therapeutic target for HCC.

## Results

### NXN expression was downregulated in HCC and associated with OS

**Fig. 1:**
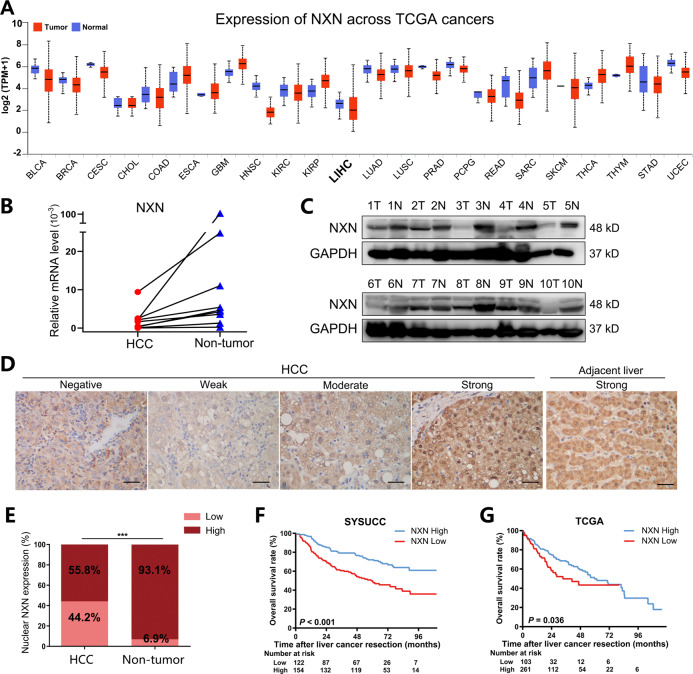
NXN was downregulated in HCC, and its low levels were associated with poor prognosis. **A** NXN mRNA expression (RNA-seq RSEM, log2(norm count +1)) in common human tumor tissues and normal tissues in the TCGA database (UCLCAN). Sample lines represent medians and quartiles. The key to all abbreviations is shown in Table S1. **B** Quantitative real-time PCR and **C** Western blot showing the expression of the NXN in 10 paired HCC and adjacent non-tumor tissues. **D** Representative images of different nucleus NXN expressions in HCC and adjacent normal liver tissues. Scale bar, 100 µm. **E** Nuclear NXN expression was downregulated in HCC tissues compared to adjacent non-tumor liver tissues (*n* = 276, paired *t*-test). ***P < 0.001, based on the Chi-squared test. **F** Kaplan-Meier analysis revealed that low NXN was associated shorter overall survival in the SYSUCC HCC cohort (*n* = 276). **G** Kaplan-Meier analysis of overall survival in TCGA cohort (*n* = 364) with high and low NXN expression.

Investigating the expression of NXN in different tumors and adjacent normal tissues can provide information about its role in cancer. Data from The Cancer Genome Atlas (TCGA) database showed that NXN mRNA expression varied among different cancer types, indicating that it might function differently in the development of diverse tumors (Fig. 1A). Analysis of the TCGA cohort showed that NXN mRNA expression was downregulated in HCC compared with normal liver tissues (*P* = 0.002; Fig. S1). Next, we detected the mRNA and protein levels of NXN in 10 HCC patients. The data confirmed that NXN was significantly lower in HCC tissues than in adjacent normal liver tissues (Fig. 1B, C). We further evaluated the expression of NXN in a tissue microarray (TMA) containing 276 paired human HCC samples (Fig. 1D). Our results showed that the expression of NXN was significantly downregulated in the nucleus of HCC tissues compared with paired adjacent liver tissues (*P* < 0.001; Fig. 1E). Kaplan-Meier analyses revealed that lower NXN expression was associated with shorter overall survival (OS) in HCC patients, both in our cohort and the TCGA cohort (*P* < 0.001 and *P* = 0.036, respectively; Fig. 1F, G).

Next, we investigated the association between NXN level and clinicopathological characteristics. We found that patients with low NXN tended to have higher aspartate transaminase (AST) levels (43.4% vs. 30.5%, *P* = 0.027), larger tumor size (6.0 vs. 4.5 cm, *P* = 0.005) and more incomplete tumor capsule (92.6% vs. 83.1%, *P* = 0.019) compared to patients with high NXN expression (Table 1). Cox proportional hazards regression analysis showed that low NXN expression was an independent unfavorable factor for OS (hazard ratio (HR) 1.963, 95% confidence interval (CI) 1.348–2.859, *P* < 0.001; Table 2). In the TCGA cohort, patients with low NXN had more vascular invasion than those with high NXN expression (44.3% vs. 30.0%, *P* = 0.017; Table S2). Notably, multivariate analyses also confirmed that NXN expression was an independent predictor for OS in HCC patients (HR 1.576, 95% CI 1.067, *P* = 0.022; Table S3). Overall, these findings demonstrated that NXN expression was downregulated in HCC tissues, and NXN was an independent positive predictive indicator for OS in HCC patients.

**Table 1:** Correlation of clinicopathological parameters and NXN expression in tumor nucleus in our cohort.

Characteristics	Low NXN (*n* = 122)	High NXN (*n* = 154)	*P* value
Age (year)	51 (41.75, 61.00)	53 (43, 60)	0.998
Gender (male/female)	102/20 (83.6/16.4)	136/18 (88.3/11.7)	0.293
HBsAg (yes/no)	113/9 (92.6/7.4)	141/13 (91.6/8.4)	0.825
ALB (≤35/>35) (g/L)	8/114 (6.6/93.4)	9/145 (5.8/94.2)	0.807
TBIL (>17.1/≤17.1) (μmol/L)	35/87 (28.7/71.3)	37/117 (24.0/76.0)	0.381
ALT (>40/≤40) (U/L)	54/68 (44.3/55.7)	67/87 (43.5/56.5)	0.900
AST (>45/≤45) (U/L)	53/69 (43.4/56.6)	47/107 (30.5/69.5)	0.027*
AFP (>400/≤400) (ng/ml)	54/66 (45/55)	52/99 (34.4/ 65.6)	0.081
Tumor number (multiple/solitary)	24/98 (19.7/80.3)	29/125 (18.8/81.2)	0.879
Tumor size (cm)	6 (4, 9)	4.5 (3, 7)	0.005*
Tumor capsule (incomplete/complete)	113/9 (92.6/7.4)	128/26 (83.1/16.9)	0.019*
Vascular invasion (yes/no)	46/76 (37.7/62.3)	56/98 (36.4/63.6)	0.900
Cirrhosis (yes/no)	88/34 (72.1/27.9)	98/56 (63.6/36.4)	0.156
TNM stage (III-IV/I-II)	14/108 (11.5/88.5)	11/143 (7.1/92.9)	0.291

*HBsAg* hepatitis B surface antigen, *ALB* albumin, *TBIL* total bilirubin, *ALT* *alanine aminotransferase*, *AST* aspartate transaminase, *AFP* alpha-fetoprotein, *TNM stage* pathological tumor-node-metastasis stage. *P < 0.05.

**Table 2:** Univariate and multivariate Cox regression analyses for overall survival in our cohort.

Variables	Univariate analysis			Multivariate analysis		
	HR	95% CI	*P* value	HR	95% CI	*P* value
NXN (low/high)	2.126	1.479–3.055	<0.001*	1.963	1.348-2.859	<0.001*
Age (year) (≤50/>50)	0.867	0.605–1.243	0.438			
Gender (male/female)	1.961	1.026–3.749	0.042*	1.932	0.990-3.733	0.053
HBsAg (yes/no)	0.839	0.451–1.560	0.578			
ALB (g/L) (≤35/>35)	1.476	0.772–2.821	0.239			
TBIL (μmol/L) (>17.1/≤17.1)	1.103	0.739–1.648	0.630			
ALT (U/L) (>40/≤ 40)	1.789	1.248–2.564	0.002*	1.349	0.888–2.050	0.161
AST (U/L) (>45/≤ 45)	2.308	1.612–3.304	<0.001*	1.619	1.055–2.484	0.027*
AFP (ng/ml) (>400/≤400)	1.339	0.930–1.928	0.116			
Tumor number (multiple/solitary)	2.012	1.339–3.022	0.001*	1.981	1.313–2.989	0.001*
Tumor size (cm) (>5/≤5)	1.529	1.065–2.194	0.021*	1.231	0.836–1.813	0.293
Tumor capsule (incomplete/complete)	1.209	0.663–2.205	0.536			
Vascular invasion (yes/no)	1.138	0.790–1.640	0.488			
Cirrhosis (yes/no)	0.963	0.875–1.061	0.449			
TNM stage (III-IV/I-II)	2.919	1.761–4.841	<0.001*			

*HR* hazard ratio, *CI* confidence interval, *NXN* expression in tumor nucleus, *HBsAg* hepatitis B surface antigen, *ALB* albumin, *TBIL* total bilirubin, *ALT* alanine aminotransferase, *AST* aspartate transaminase, *AFP* alpha-fetoprotein, *TNM stage* tumor-node-metastasis stage. *P < 0.05.

### NXN suppressed HCC cell proliferation, migration and invasion in vitro

**Fig. 2:**
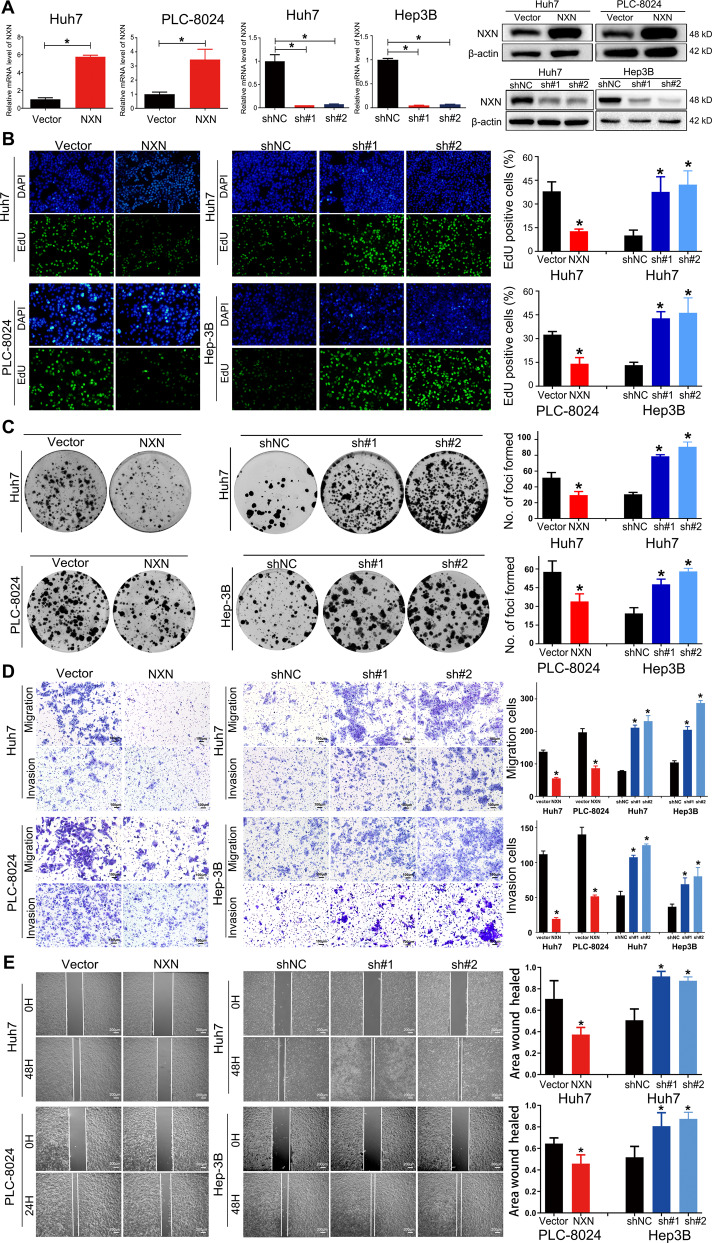
NXN suppressed the proliferation, migration and invasion of HCC cells in vitro. **A** The efficiency of NXN overexpression and knockdown was measured by quantitative real-time PCR (left) and Western blot (right) assays. NXN suppressed the cell growth of HCC cells, evaluated by EdU (**B**) and colony formation assays (**C**). Nuclei of all cells were stained with blue, and nuclei of cells with high DNA replication activities (EdU-positive cells) were stained with green. NXN suppressed the migration and invasion of HCC cells, evaluated by Transwell (**D**) and wound healing assay (**E**). Statistical results are presented as mean ± SD (from triplicates), and significance was determined by Student’s *t*-test (*P < 0.05).

To evaluate the biological functions of NXN in HCC cells, the expression of NXN in HCC cell lines was first examined (Fig. S2A). According to the endogenous expression level of NXN in HCC cells, we stably overexpressed NXN in Huh7 and PLC-8024 cells with plasmid and knocked down NXN in Huh7 and Hep3B cells using two shRNAs for further study (Fig. 2A).

Since NXN expression was negatively associated with tumor size, the regulatory effect of NXN in HCC cell proliferation was analyzed. As shown in Fig. 2B, EdU staining of the NXN knockdown cells exhibited a remarkable increment in the percentage of proliferating cells compared with controls, while opposing effects on cell proliferation were seen in the overexpression experiment. CCK-8 and colony formation assays also confirmed that overexpression of NXN significantly suppressed HCC cell growth, while knockdown of NXN promoted HCC cell growth abilities (Fig. S2B, C, respectively).

Considering that NXN expression was correlated with tumor capsule and vascular invasion, the functions of NXN in HCC cell migration and invasion were explored. Transwell migration, Matrigel invasion and wound healing assays revealed that, compared with vector cells, the ectopic expression of NXN suppressed HCC cell migration. In contrast, inhibition of NXN enhanced the motility of HCC cells (Fig. 2D, E). Taken together, NXN contributed to the inhibition of HCC cell proliferation, migration and invasion.

### NXN inhibited HCC cell growth and metastasis in vivo

**Fig. 3:**
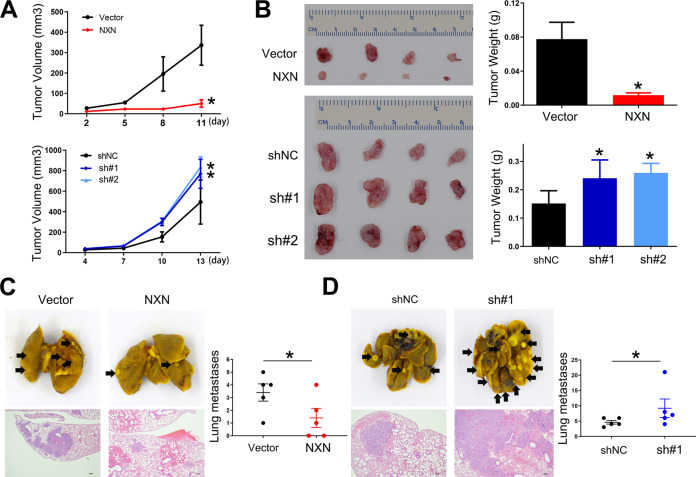
NXN suppressed the proliferation and metastasis of HCC cells in vivo. NXN overexpression suppressed the proliferation of HCC cells in nude mice. Tumors volume (**A**) and tumor weight (**B**) after subcutaneous injection of indicated Hepa1-6 cells. Four mice per group. NXN overexpression suppressed metastasis of HCC cells in nude mice. Representative images of metastatic lung tumors (gross and HE staining) and quantification in NXN overexpression (**C**) and NXN knockdown group (**D**) after tail vein injection. Scale bar, 100 µm. Five mice per group. *P < 0.05, Student’s *t*-test.

To determine whether NXN had the same effect on HCC progression in vivo, Hepa1–6 cells with NXN enforced or inhibited expression were used in mice models. After subcutaneously injecting the mice with the indicated cells (Fig. S3A), tumors in the NXN overexpression group were found to be significantly smaller than in the vector group (Fig. 3A). The tumor weight of the NXN ectopic expression group was also significantly decreased compared with the vector group (Fig. 3B). Further, NXN knockdown was associated with an increase in the volume and weight of tumors compared with the control group.

Next, we generated a lung metastasis mouse model via tail vein injection of the indicated Hepa1–6 cells (Figure S3B). Fewer metastatic lung nodules were observed in mice injected with NXN overexpression cells than those injected with vector cells (Fig. 3C). Further, more metastatic lung nodules were observed in mice injected with the NXN knockdown cells than in those injected with control cells (Fig. 3D). Collectively, these results indicated that NXN suppressed HCC growth and metastasis in vivo.

### NXN reduced EMT in HCC cells

**Fig. 4:**
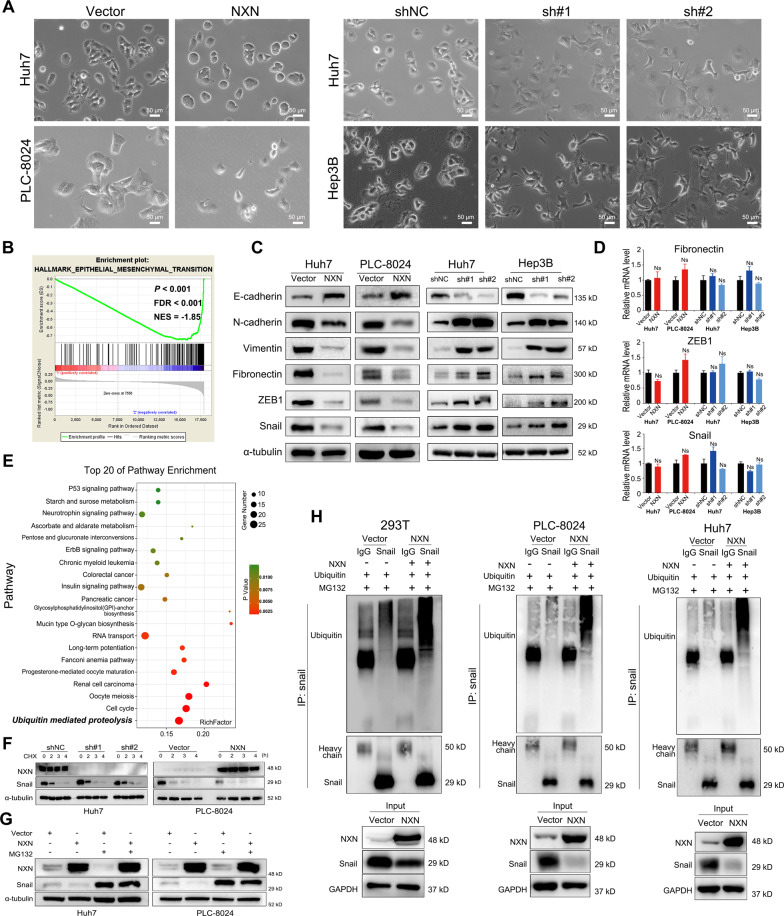
NXN suppressed EMT and decreased Snail protein stability. **A** Differential expression of NXN affects cell morphology. Phase-contrast images (× 200) of indicated HCC cells. Scale bar, 50 µm. **B** Gene set enrichment analysis (GSEA) of transcriptome data in the TCGA database revealed that levels of NXN were negatively correlated with epithelial-mesenchymal transition (EMT). NES, normalized enrichment score; FDR, false-discovery rate. **C** Western blot revealed that NXN negatively regulated the protein expression of mesenchymal markers in HCC cells. **D** Quantitative real-time PCR analysis showed that NXN expression did not affect the mRNA levels of transcription factors (Fibronectin, ZEB1 and Snail). Data were presented as the mean ± SD. Ns, no statistical significance. **E** The down-regulation of NXN in HCC activated the ubiquitin-mediated proteolysis signaling pathway. Top 20 statistics of KEGG pathway enrichment based on the differentially expressed genes (DEGs) after NXN knockdown in Hep3B cells. In the scatter plot, RichFactor was the ratio of DEG numbers noted in this pathway term to all gene numbers noted in this pathway term, indicating intensiveness. *P*-value ranges from 0 to 1, and a lower *P*-value represents greater intensity. The ubiquitin-mediated proteolysis signaling pathway was one of the most regulated biofunctions upon NXN knockdown. **F** The half-life of Snail in Huh7 with NXN knockdown was significantly prolonged compared to control cells. The half-life of Snail in NXN overexpressed PLC-8024 cells was markedly reduced compared to control cells. The cells were treated with cycloheximide (CHX) at indicated times; then, Snail levels were analyzed by Western blot. **G** Downregulation of Snail in NXN overexpressed Huh7 and PLC-8024 cells were restored after the treatment of proteasome inhibitor (MG132). **H** NXN overexpression enhanced ubiquitination of Snail in 293 T, PLC-8024 and Huh7 cells.

EMT plays crucial roles in tumor metastasis. When the expression of NXN was knocked down in Huh7 and Hep3B cells, we found that the morphology of some cells transitioned from an epithelial-like form to a spindle-shaped or elongated, mesenchymal form, indicating that NXN may suppress HCC progression by inhibiting EMT (Fig. 4A).

By Gene Set Enrichment Analysis (GSEA) in the TCGA dataset, we found that EMT-related genes were significantly enriched in the HCC patients with low NXN expression (*P* < 0.001, normalized enrichment score (NES) = −1.85; Fig. 4B). Western blot confirmed that overexpressing NXN in Huh7 and PLC-8024 cells upregulated the expression of epithelial markers E-cadherin and reduced the expression of mesenchymal markers N-cadherin and Vimentin as well as that of EMT-TFs Fibronectin, ZEB1 and Snail (Fig. 4C). In contrast, NXN knockdown downregulated the expression of E-cadherin and increased that of N-cadherin, Vimentin, Fibronectin, ZEB1 and Snail in Huh7 and Hep3B cells. However, NXN did not affect the mRNA level of the EMT-TFs (Fig. 4D). These results indicated that NXN inhibited the progression of HCC cells, at least partly through post-transcriptional regulation in EMT.

### NXN promoted the degradation of Snail protein by ubiquitination

To elucidate the molecular mechanisms of NXN in regulating HCC metastasis, we performed genomic expression profiling of Hep3B cells with NXN knockdown or control. Kyoto Encyclopedia of Genes and Genomes (KEGG) pathway enrichment analysis identified that the ubiquitin-mediated proteolysis signaling pathway was significantly enriched (Fig. 4E). As the ubiquitin-proteasome degradation pathway is the main mechanism controlling the Snail protein level, we further examined the interaction between NXN and Snail [10, 19, 20]. We first examined whether NXN had an effect on promoting Snail degradation. NXN-depletion-Huh7 cells were treated with Cycloheximide (CHX) to block new protein synthesis. The resulting cells were collected at various time points (0, 2, 3, 4 h), and Western blot was used to examine the protein level of Snail. Indeed, Snail protein stability was enhanced in NXN-shRNA cells than in control cells, while overexpression of NXN in PLC-8024 markedly induced degradation of Snail protein (Fig. 4F).

As Snail protein degradation is mediated by the ubiquitin-proteasome pathway, to pinpoint the in-depth mechanism of NXN-induced Snail degradation, we treated NXN overexpression cells with a proteasome inhibitor (MG132). We found that MG132 could significantly restore Snail protein levels in NXN overexpressed Huh7 and PLC-8024 cells, indicating that the ubiquitin-proteasome pathway was involved in the process of Snail degradation induced by NXN (Fig. 4G).

We evaluated Snail ubiquitination in NXN-overexpressed 293 T, PLC-8024 and Huh7 cells to further validate these findings. As expected, Snail ubiquitination was significantly increased when NXN was overexpressed (Fig. 4H). Taken together, these data indicated that NXN decreased Snail stability by promoting the ubiquitin–proteasome-mediated degradation.

### NXN promoted Snail ubiquitination by competing with DUB3

**Fig. 5:**
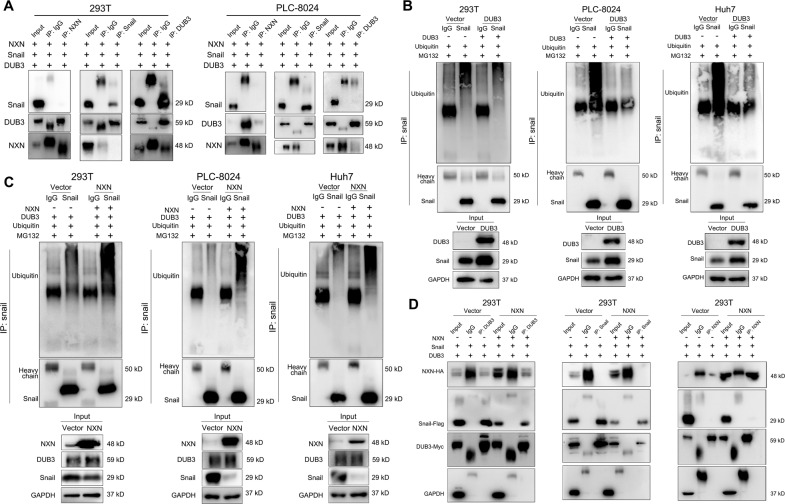
NXN interrupted the association between DUB3 to bind with Snail and inhibited deubiquitylation of Snail by DUB3. **A** Co-immunoprecipitation (co-IP) was performed to detect the interaction between NXN, Snail and DUB3 in 293 T and PLC-8024 cells. NXN did not interact with Snail protein, whereas NXN or Snail could co-immunoprecipitated with DUB3. **B** DUB3 overexpression reduced the ubiquitinated Snail in 293 T, PLC-8024 and Huh7 cells. **C** NXN overexpression reduced the DUB3-mediated Snail deubiquitylation in 293T, PLC-8024 and Huh7 cells. Cells were transfected with indicated plasmid. After 48 h, the cells were incubated with MG132 (10 mM) for 4 h, then the cell lysates were immunoprecipitated with anti-Flag, and the immunocomplexes were analyzed by Western blot using an anti-ubiquitin antibody. **D** NXN overexpression reduced the interaction between DUB3 and Snail. Total lysates of cells with or without NXN overexpression were subjected to IP with anti-DUB3 or anti-Snail Ab, followed by Western blot using the indicated antibodies. The cells were treated with MG132 to inhibit the proteasome.

Given that Snail could be deubiquitinated and stabilized by DUB3, we explored the relationships among NXN, Snail and DUB3 in HCC cells [9, 19]. Co-IP assays were performed to check the interaction among NXN, Snail and DUB3 in 293 T cells (Fig. 5A). IP results showed that NXN and Snail could co-immunoprecipitate with DUB3, whereas NXN did not interact with Snail protein. Consistently, reciprocal IP with Snail antibody did not pull down NXN. These results suggested that NXN regulated the stability of Snail through the connection of DUB3.

We further verified the functional significance of DUB3 in Snail deubiquitylation. After overexpressing DUB3 in 293 T, PLC-8024 and Huh7 cells, the level of Snail protein ubiquitination was significantly reduced (Fig. 5B). In DUB3 overexpressed cells, the ubiquitination level of Snail was significantly increased in the presence of enforced expression of NXN (Fig. 5C). Next, we found that overexpression of NXN could significantly reduce the binding of Snail and DUB3 (Fig. 5D).

Collectively, these data confirmed that NXN could competitively interrupt the association between DUB3 to bind with Snail and inhibited the deubiquitylation of Snail by DUB3, thereby stabilizing Snail and enhancing HCC metastasis.

### Snail was required for NXN to suppress HCC cell aggressiveness

**Fig. 6:**
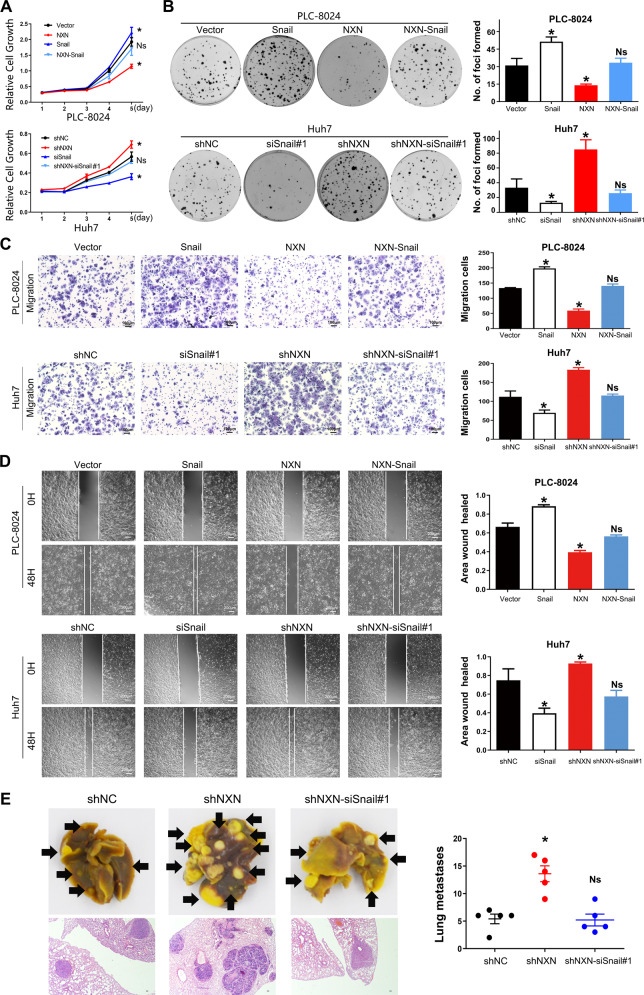
The inhibitory effect of NXN on HCC cells proliferation and metastasis was rescued by the overexpression of Snail. Overexpression of Snail attenuated NXN induced suppression of HCC cell growth, evaluated by CCK8 (**A**) and colony formation assays (**B**). Overexpression of Snail attenuated NXN induced suppression of HCC cell migration, evaluated by Transwell (**C**) and wound healing assay (**D**). Depletion of Snail in NXN knockdown group reversed NXN deficiency-induced promotion of HCC metastasis in nude mice. **E** Representative images and quantification of metastatic lung tumors (gross and HE staining after tail vein injection. Scale bar, 100 µm. Five mice per group. Statistical results were presented as mean ± SD (from triplicates), and significance was determined by Student’s *t*-test (*P < 0.05).

Next, we examined whether the effects of NXN on the suppression of HCC were Snail-dependent. Snail was depleted (siSnail) in NXN silenced Huh7 cells and was also ectopically expressed in NXN-overexpressed PLC-8024 cells (Fig. S4A). CCK-8 and colony formation assays results showed that forced expression of Snail significantly reversed NXN-overexpression-induced inhibition of cell proliferation (Fig. 6A, B). Depletion of Snail also reversed NXN-silencing and promoted HCC cell migration and metastasis (Fig. 6C–E). By detecting the downstream target genes of Snail in transcriptional levels, we demonstrated that the effects of NXN on the suppression of HCC were Snail-dependent (Fig. S4C). Together, these data demonstrated that Snail was an essential functional target of NXN in HCC cells.

**Fig. 7:**
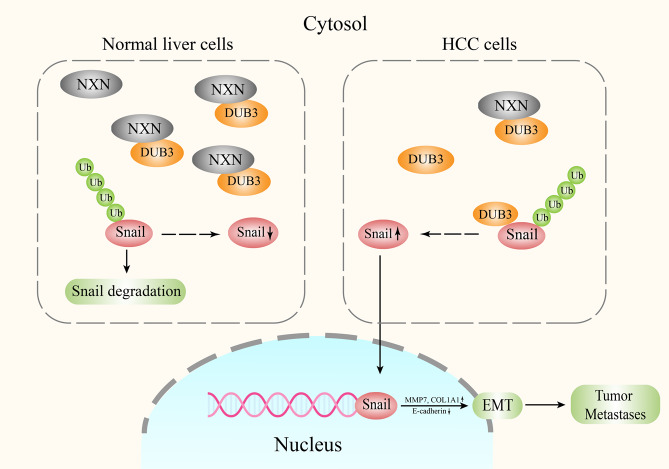
Schematic illustration depicting the role of NXN in suppressing tumor metastasis through promoting degradation of Snail. In normal liver cells, NXN promotes the ubiquitin-proteasome-mediated degradation of Snail. In HCC cells, the reduction of NXN decreases the interaction between NXN and DUB3 and results in the increased combination of Snail and DUB3. The increased interaction between DUB3 and Snail activates EMT and ultimately contributes to HCC metastases.

Overall, our study demonstrates that NXN, DUB3 and Snail complex functioned as an important regulatory mechanism of HCC progression and provides a potential therapeutic approach in the intervention of HCC metastasis (Fig. 7).

## Discussion

Metastasis is one of the major obstacles leading to poor prognosis in HCC patients [21–23]. Elucidating the underlying mechanism of HCC metastasis is vital for developing novel therapeutics. In this present study, we found that NXN could affect HCC metastasis. NXN was commonly downregulated in HCC tissues, and its low expression was associated with larger tumor size, incomplete tumor capsule and unfavorable OS in HCC patients. Moreover, downregulation of NXN expression contributed to the transition to a mesenchymal phenotype and enhanced the growth and metastasis of HCC cells both in vitro and in vivo. Mechanistically, NXN suppressed HCC growth and metastasis by regulating Snail ubiquitination and degradation.

NXN has been widely documented in regulating physiological processes [13, 24, 25]. Knockout of NXN could result in skeletal defects, cardiovascular defects and perinatally lethal [14]. Dysregulation of NXN has been indicated to increase the expression of fibrogenic genes [15, 16]. Moreover, deficiency of NXN was observed to result in the progression of alcoholic liver disease (ALD) [26, 27]. However, NXN overexpression partially reversed these alterations and reduced liver injury, indicating that NXN played an important role in maintaining tissue homeostasis. In agreement with the protective effect of NXN in normal tissues, our results showed that NXN might function as a tumor suppressor in HCC because it was downregulated in HCC tissues compared to normal tissues and that low NXN expression was associated with poor prognosis for HCC patients.

Although the physiological functions of NXN have been studied in detail, the function of NXN in cancer remains largely unknown. One study identified that NXN expression was reduced in colon tumor tissue samples and associated with colorectal cancer risk [28]. Other researchers found that NXN expression was upregulated in retinoic acids-treated breast cancer cell line MCF-7 compared with untreated MCF-7 cells [18]. In this study, we demonstrated that NXN could markedly inhibit the proliferation and metastasis of HCC in vitro and in vivo, indicating the potential tumor-suppressive effect of NXN in HCC development. In addition, NXN dramatically decreased the level of Snail as well as EMT progression. Herein, we speculated that the molecular mechanisms of NXN-mediated suppression of cell proliferation and metastasis in HCC might be involved in the regulation of the Snail-dependent EMT axis.

Cancer cell EMT, featured by enhancing cancer cell dissemination, contributes to HCC metastasis [29, 30]. It has been widely reported that Snail played crucial roles in hepatocarcinogenesis and metastasis [31, 32]. Snail is highly susceptible to ubiquitination and proteasomal degradation controlled by ubiquitin E3 ligases, including FBXL14, SPSB3 and TRIM50 [20, 33, 34]. Ubiquitination can be reversed by deubiquitinases (DUBs). Most recently, DUB3, PSMD14 and USP29 have been reported to deubiquitinate and stabilize Snail [9, 10, 35]. As an enzyme, NXN might exhibit its functions by protein-protein interactions network. It was reported that NXN regulated ethanol-associated ALD progression by binding to FLII/actin or MYD88 [26, 27]. By expelling KLHL12 from DVL protein, NXN resulted in the inhibition of Dvl ubiquitination to suppress the Wnt/β-catenin signaling [14]. Similar to the regulatory mechanism in previous studies, we identified that NXN competitively interrupted the association between DUB3 to bind with Snail and inhibited deubiquitylation of Snail by DUB3, thereby stabilizing Snail and enhancing HCC metastasis. These findings spotlighted that NXN acted as a novel and key modulator for Snail in HCC, suggesting that it may serve as a potential biomarker and therapeutic target for HCC.

In summary, our study, for the first time, found that NXN could affect HCC metastasis. NXN, DUB3 and Snail complex functioned as an important regulatory mechanism of HCC progression. These findings could be used as a potential therapeutic approach in the intervention of HCC metastasis.

## Materials and methods

### Human tissue specimens

A total of 276 pairs of pathologically confirmed HCC and adjacent non-tumor liver tissues were obtained from patients who received hepatectomy at the Sun Yat-sen University Cancer Center (SYSUCC; Guangzhou, China) from April 2008 to September 2014. The median follow-up time was 76 months. Written informed consent was obtained from each patient. This study complied with the 1975 Declaration of Helsinki standards, and the experiments were approved by the Ethics Committee of SYSUCC (No. L102012020090M).

### Immunohistochemistry (IHC)

HCC specimens were selected and re-embedded into new paraffin blocks for TMA. The TMA blocks were cut into 4 μm sections and underwent IHC staining. The rabbit polyclonal antibodies used were anti-NXN (1:100; proteintech, USA). Based on IHC, positive staining was quantified and classified into 4 categories: ≤25% for 1; 26 to 50% for 2; 51 to 75% for 3; and ≥76% for 4. Staining intensity was graded as negative (scored as 0), weak (1), moderate (2) or strong (3). Two pathologists independently reviewed all scores, and expression levels were defined by the sum of the grades for the percentage of positive staining and intensity. The median of the IHC score was chosen as the cut-off value for the high (>6) and low (≤6) NXN groups.

### Cell lines and cell culture

HCC cell lines (Huh7, Hep3B, Hep-G2, PLC-8024, MHCC-97H, SK-Hep1 and Hepa1–6) and human embryonic kidney cells (293 T cells) were purchased from the Cell Bank of Shanghai Institute of Biological Science (SIBS, CAS, Shanghai, China) with STR (short tandem repeat) appraisal certificates. Cells were maintained in Dulbecco’s Modified Eagle medium (DMEM; ThermoFisher, USA) supplemented with 10% fetal bovine serum (FBS; Gibco, Califonia, USA) at 37 °C in 5% CO2.

### Cell proliferation assays

Three experiments were performed to analyze the cell proliferation ability. EdU cell proliferation staining was performed using an EdU kit (C0071S; Beyotime Biotechnology, China). Briefly, indicated cells were incubated with the EdU buffer for 3 h, fixed by 4% polyformaldehyde and stained the nuclear with Hoechst. The results were observed and captured using the fluorescence microscope (Olympus Corporation, Tokyo, Japan).

In the Cell Counting Kit-8 (CCK-8; DojinDo, Japan) assay, 1000 cells were seeded in 96-well plates with six repeated wells for each experimental condition. Two hours after replacing the supernatant with a fresh medium containing CCK-8 reagent in a 10:1 ratio, the absorbance was measured by Biotek Epoch 2 machine (BioTek, Winooski, USA) at 450 nm.

For the colony formation assay, 500–800 indicated stable cells were plated per well in 6-well plates. Fourteen days later, the cells were fixed in methanol and stained with 0.1% crystal violet. The colonies were photographed and counted to contrast with each other.

### Cell migration and invasion assays

Transwell chamber (8 μm pores, Costar, Kennebunk, USA) with polycarbonate membranes was used in the migration assay, and a chamber with matrigel (Costar, Kennebunk, USA) was used in the invasion assay. Then, 5 × 10^4^ to 1.5 × 10^5^ indicated cells for the migration assay and 8 × 10^4^ to 2 × 10^5^ indicated cells for the invasion assay were plated in the upper chamber in serum-free medium. Fresh media containing 20% FBS was placed in the bottom chamber. After 12–48 h, the cells were stained and photographed under a microscope.

For scratch wound healing assay, cells were plated in six-well plates and incubated in the regular condition until the cells reached the full confluence of the plates. A wound was created using a sterile 100 µL pipette tip, and the detached cells were removed by phosphate buffer saline (PBS). Then, the cells were incubated with serum-free DMEM for an indicated time. Images at 0, 24, and 48 h after scratching were taken, and the closure of the wound area was evaluated using ImageJ software.

### In vivo xenograft model

All animal research procedures were performed according to the Animal Care and Use Ethics Committee of SYSUCC, and careful attention was paid to minimizing the animals’ suffering. Male nude mice (Guangdong Medical Lab Animal Center) were purchased at 3–4 weeks of age and maintained under specific-pathogen-free (SPF) conditions.

To evaluate the inhibitory effect of NXN on tumor growth in vivo, 200 µL Hepa1–6 cells (2 × 10^6^ cells) were subcutaneously injected into mice. The body weight and tumor size were monitored every three days. Eleven or thirteen days after inoculation, all mice (*n* = 4 per group) were euthanized for further analyses. Tumor volume (V) was measured using the formula: V = (L × W^2^)/2 (L = length; W = width).

To establish the lung metastasis model, 1 × 10^6^ Hepa1–6 cells were injected into the tail veins of mice (*n* = 5 per group). After fifteen days, the mice were sacrificed, and the tumor nodules formed on the lung surfaces were counted.

### Co-immunoprecipitation (Co-IP)

For the Co-IP assay, cells were lysed with RIPA lysis buffer (Thermo Fisher Scientific). Primary anti-NXN or anti-IgG (negative control) antibodies were incubated with the lysates at 4 °C overnight. Protein A/G-Agarose beads (Thermo Fisher Scientific) were added to the immune complexes for recovery. After that, the immunoprecipitants were washed and collected. Finally, the immunoprecipitants were subjected to Western blot analysis.

### Statistical analysis

The experiments were repeated at least three times independently, and the measured data were represented as the mean ± SD. Binary variables were compared by the Chi-squared test. The Student’s *t*-test or the Mann-Whiney U test was used to compare the values between subgroups. Survival curves were constructed using the Kaplan-Meier method and analyzed by the log-rank test. Significant prognostic factors founded by univariate analysis were entered into a multivariate analysis using the Cox proportional hazards model. All analyses were two-sided, and *P* values less than 0.05 were considered significant. Statistical analyses were performed using the Statistical Package for Social Sciences version 25 (SPSS Inc., Chicago, IL, USA) and GraphPad Prism 7.0 software (GraphPad, Inc., La Jolla, CA, USA).

## Supplementary information

checklist table

Original Data File

supplementary figure legends and tables

Figure S1. NXN mRNA expression in human tumor tissues and normal tissues in TCGA database.

Figure S2. Overexpression of NXN inhibited proliferation of HCC cells in vitro.

Figure S3. Overexpression of NXN inhibited proliferation and metastasis of HCC cells in vivo.

Figure S4. The promoting effect of NXN depletion on HCC cells metastasis was rescued by the knock-down of Snail.

## Data Availability

The RNA-Seq data are available at the Gene Expression Omnibus (GSE208734). Other datasets used and/or analyzed during the current study are available from the corresponding author on reasonable request.
