# Supplementary material for: NXN suppresses metastasis of hepatocellular carcinoma by promoting degradation of Snail through binding to DUB3
Source: Cell Death Dis. 2022 Aug 4;13(8):676. doi: 10.1038/s41419-022-05135-7 (PMC9352874; doi:10.1038/s41419-022-05135-7)

Figure 1C

NXN:
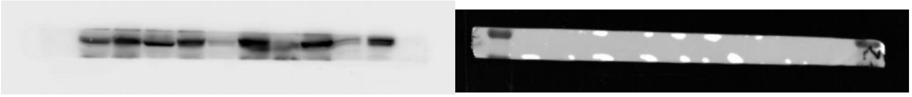

GAPDH:
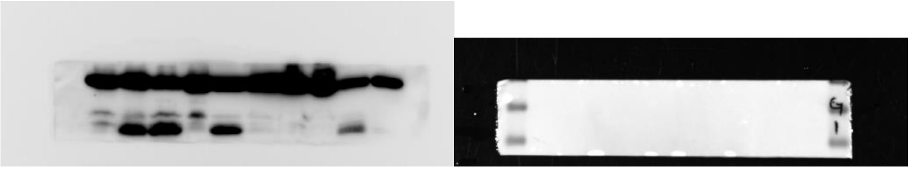

NXN:
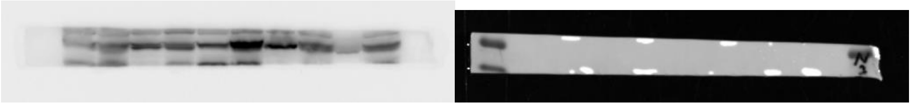

GAPDH:
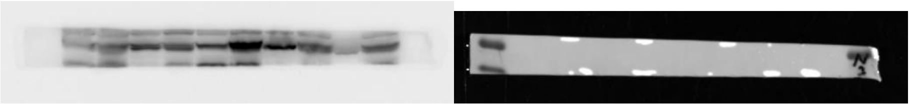

Figure 2A

NXN in Huh7-oe:

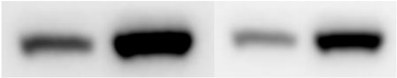

NXN in PLC-8024-oe:

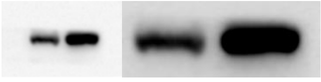

NXN in Huh7-sh:

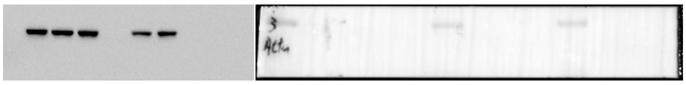

NXN in Hep-3B-sh:

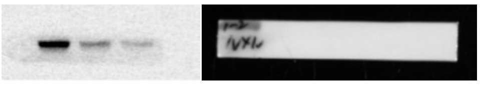

β-actin in Huh7-oe:

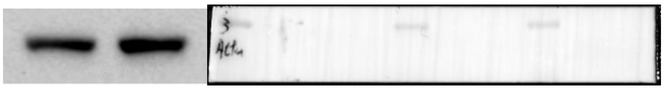

β-actin in PLC-8024-oe:

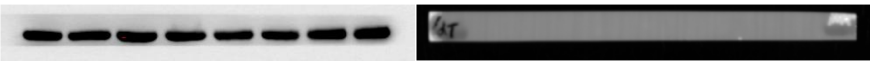

β-actin in Huh7-sh:

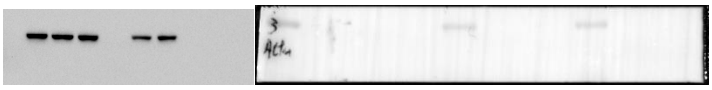

β-actin in Hep-3B-sh:

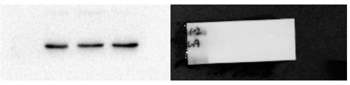

Figure 4C

For Huh7-oe:

E-cadherin: 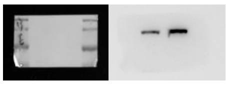

N-cadherin: 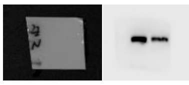

Vimentin: 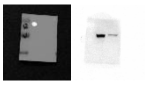

Fibronectin: 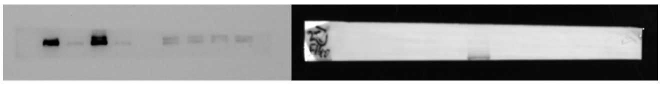

ZEB1: 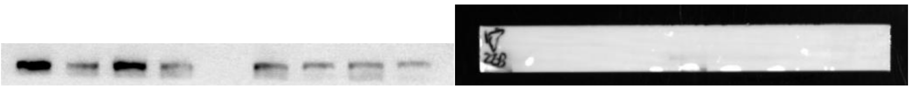

Snail: 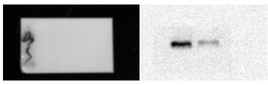

α-tubulin: 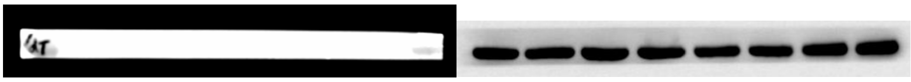

For PLC-8024-oe:

E-cadherin: 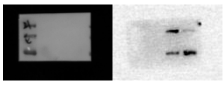

N-cadherin: 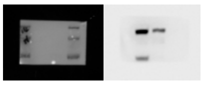

Vimentin: 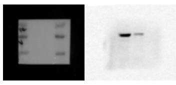

Fibronectin: 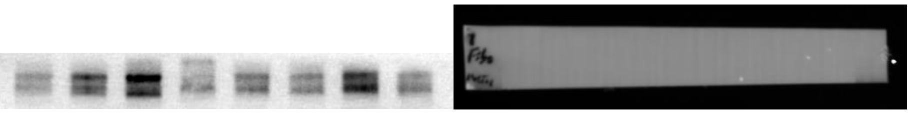

ZEB1: 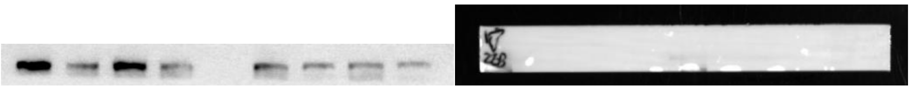

Snail: 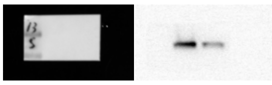

α-tubulin: 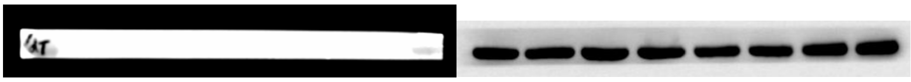

For Huh7-sh:

E-cadherin: 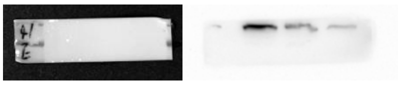

N-cadherin: 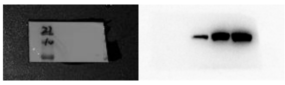

Vimentin: 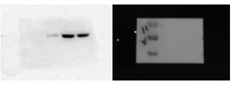

Fibronectin: 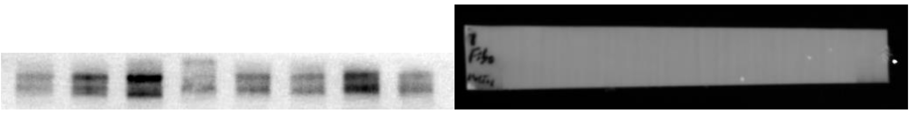

ZEB1: 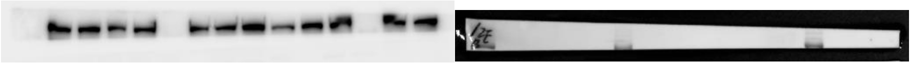

Snail: 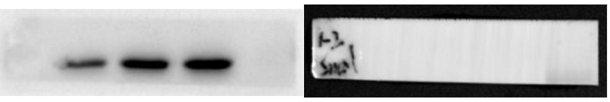

α-tubulin: 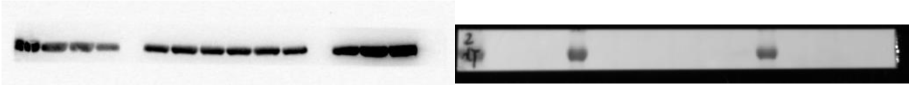

For Hep-3B-sh:

E-cadherin: 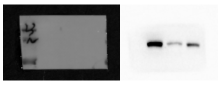

N-cadherin: 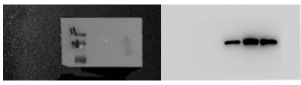

Vimentin: 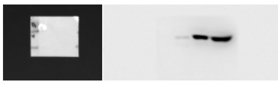

Fibronectin:

ZEB1: 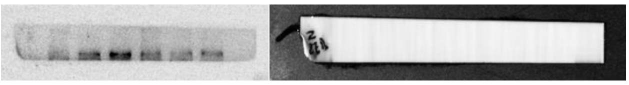

Snail: 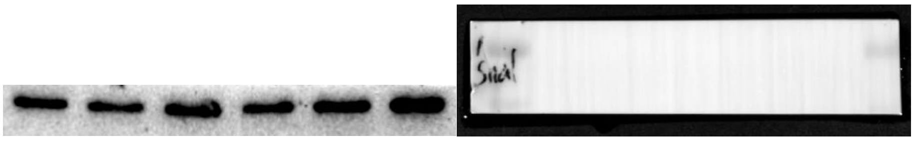

α-tubulin: 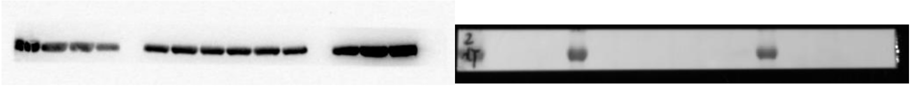

Figure 4F

Huh7-sh:

NXN: 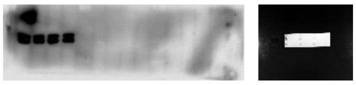

Snail: 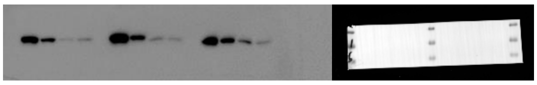

a-tubulin: 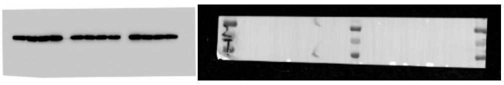

PLC-8024-oe:

NXN: 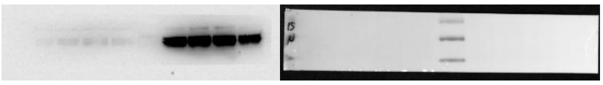

Snail: 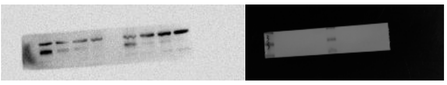

a-tubulin: 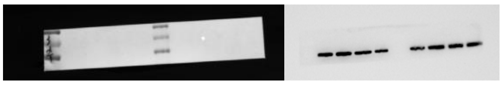

Figure 4G

Huh7-oe:

NXN:
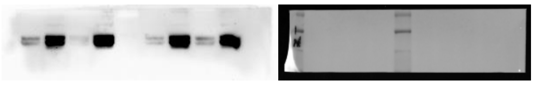

Snail:
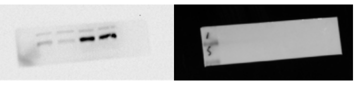

a-tubulin:
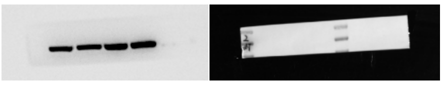

PLC-8024-oe:

NXN:
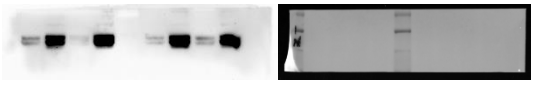

Snail:
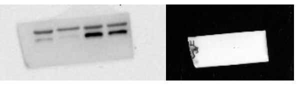

a-tubulin:
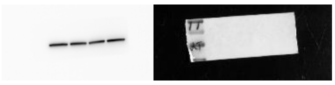

Figure 4H

293T:

IP: snail

Ub:

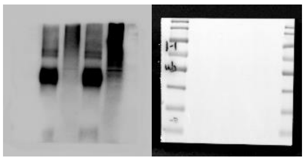

Snail:

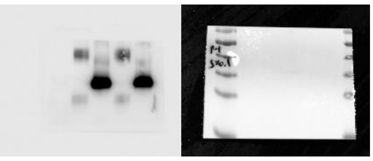

Input

NXN:

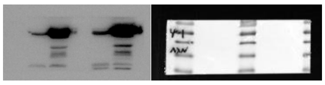

Snail:

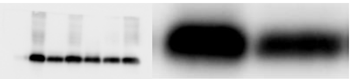

GAPDH:

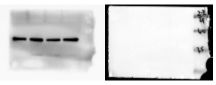

PLC-8024:

IP: snail

Ub:

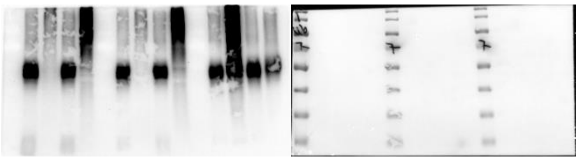

Snail:

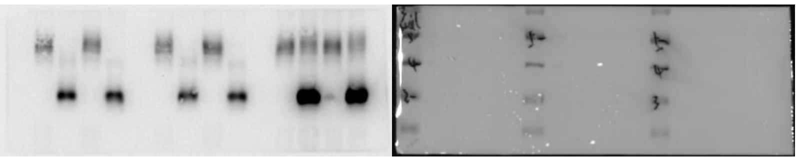

Input

NXN:

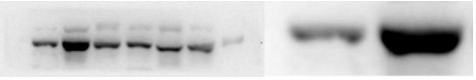

Snail:

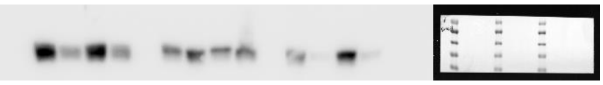

GAPDH:

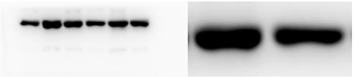

Huh7:

IP: snail

Ub:

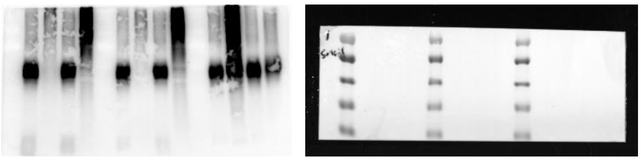

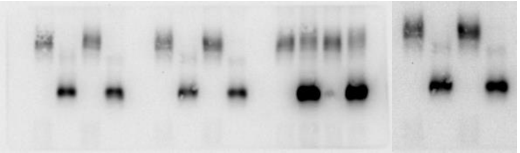

Snail:

Input

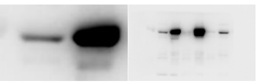

NXN:

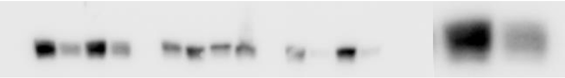

Snail:

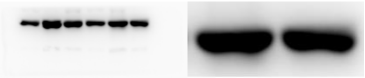

GAPDH:

Figure 5A

293T:

IP: NXN

Snail: 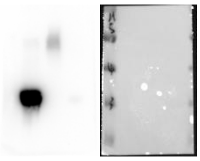

DUB3: 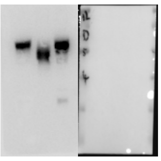

NXN: 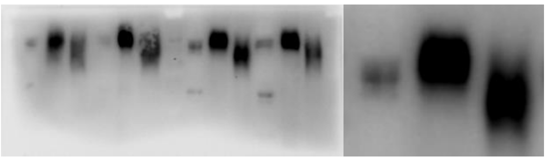

IP: snail

Snail: 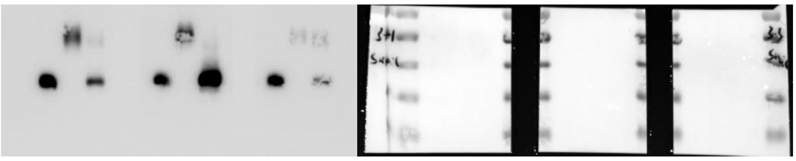

DUB3: 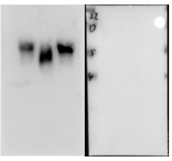

NXN: 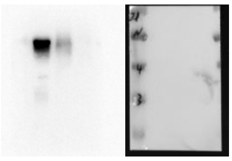

IP: DUB3

Snail: 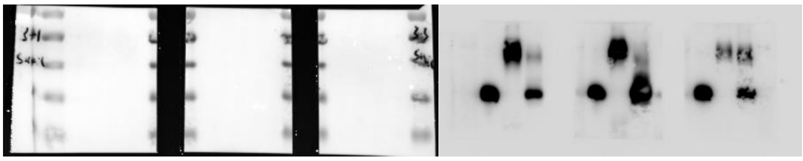

DUB3: 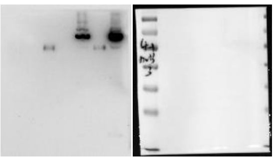

NXN: 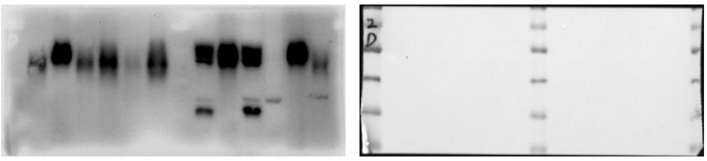

PLC-8024

IP: NXN

Snail: 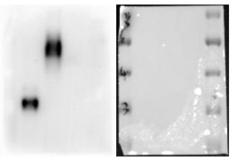

DUB3: 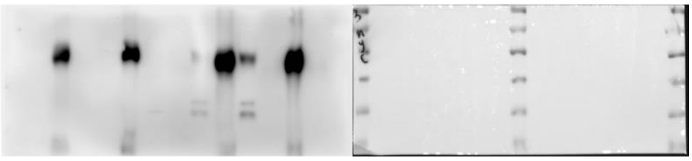

NXN: 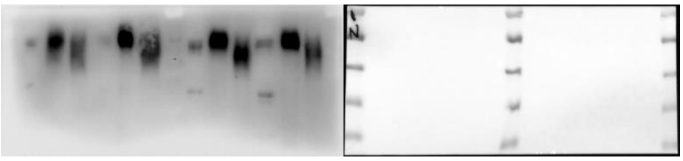

IP: snail

Snail: 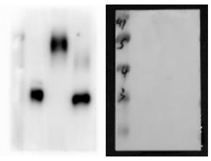

DUB3: 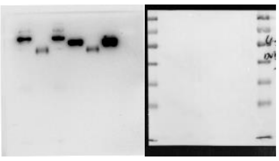

NXN: 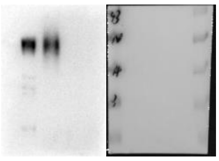

IP: DUB3

Snail: 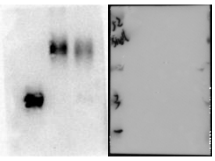

DUB3: 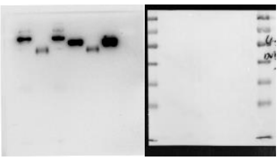

NXN: 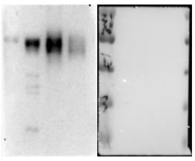

Figure 5B

293T

IP: snail

Ub: 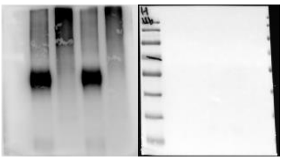

Snail: 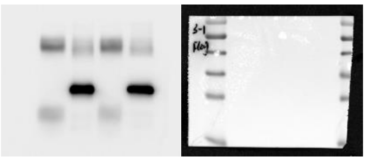

Input

DUB3: 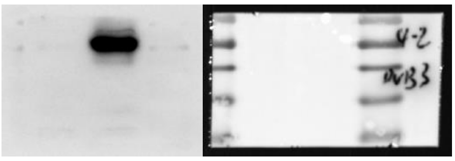

Snail: 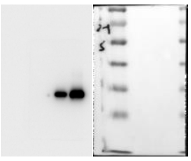

GAPDH: 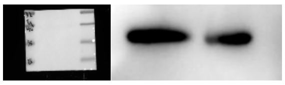

PLC-8024

IP: snail

Ub: 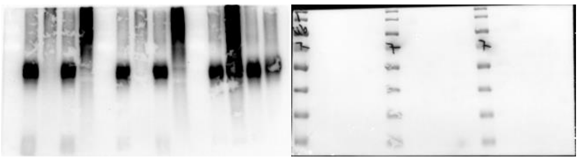

Snail: 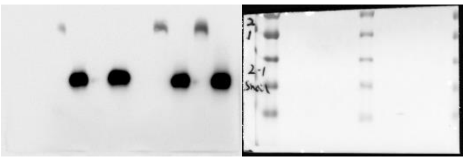

Input

DUB3: 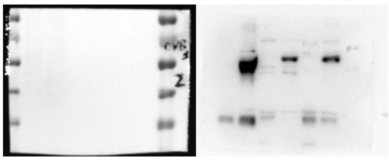

Snail: 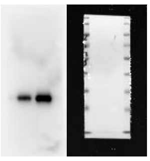

GAPDH: 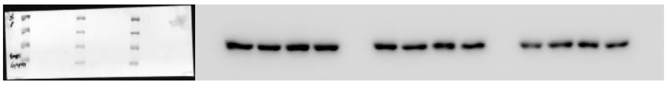

Huh7:

IP: snail

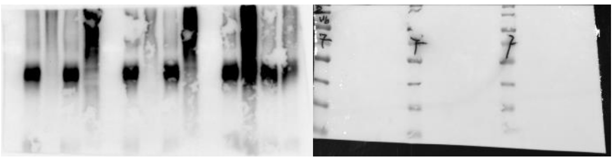

Ub:

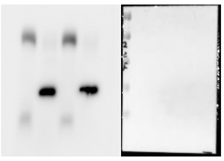

Snail:

Input

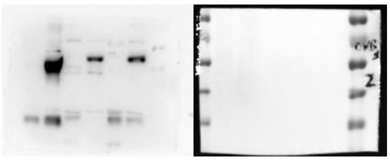

DUB3:

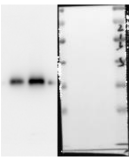

Snail:

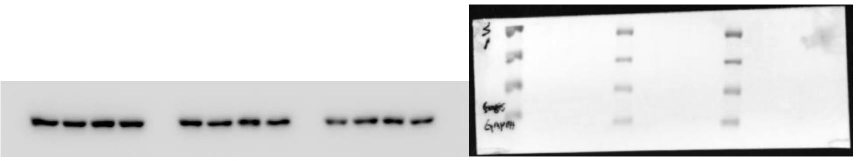

GAPDH:

Figure 5C

293T

IP: snail

Ub:

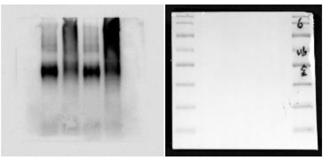

Snail:

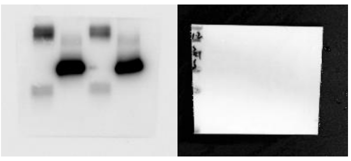

Input

NXN:

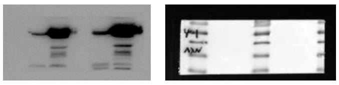

Snail:

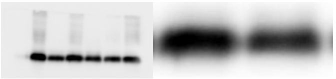

GAPDH:

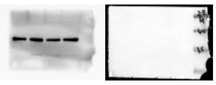

PLC-8024

IP: snail

Ub:

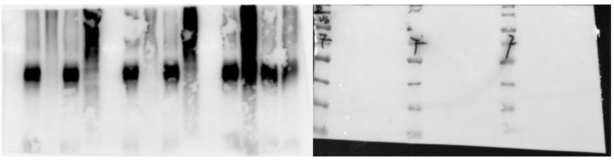

Snail:

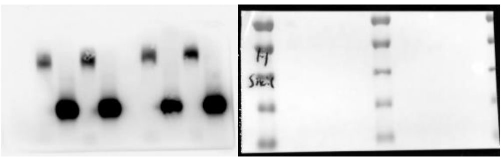

Input

NXN:

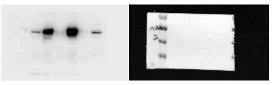

Snail:

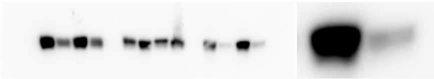

GAPDH:

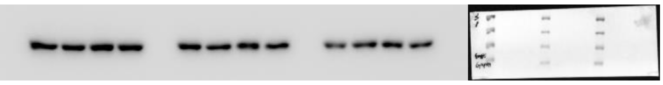

Huh7

IP: snail

Ub:

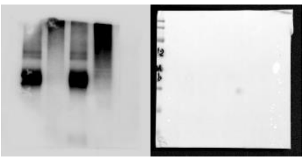

Snail:
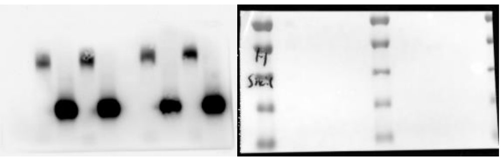

Input

NXN:
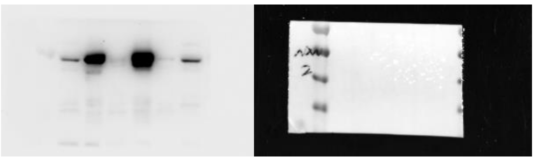

Snail:
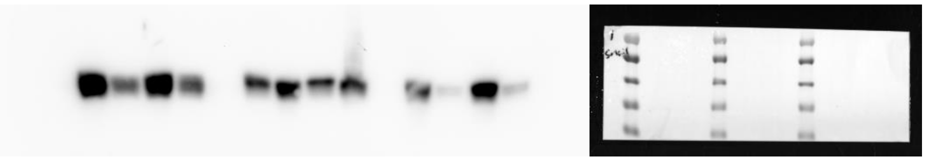

GAPDH:
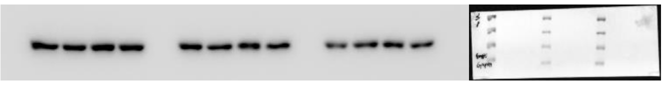

Figure 5D

293T

IP: DUB3

NXN: 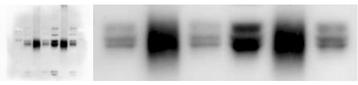

Snail: 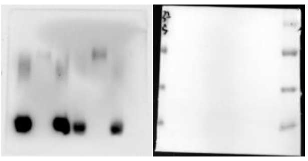

DUB3: 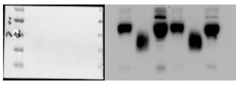

GAPDH: 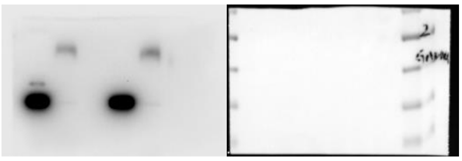

IP: Snail

NXN: 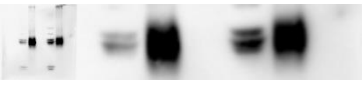

Snail: 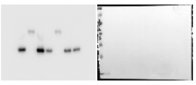

DUB3: 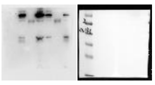

GAPDH: 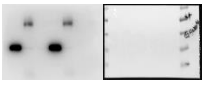

IP: NXN

NXN: 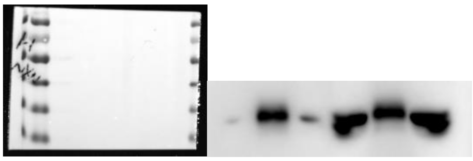

Snail: 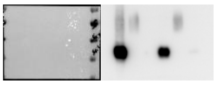

DUB3: 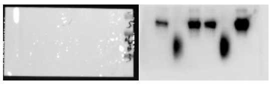

GAPDH: 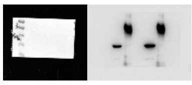

Figure S2A

NXN:

β-actin:

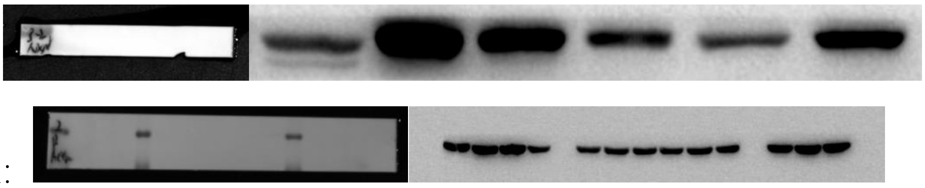

Figure S4A

PLC-8024

Snail: 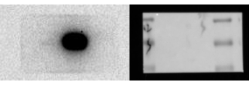

a-Tubulin: 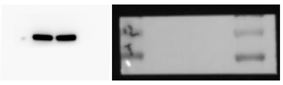

Huh7

Snail: 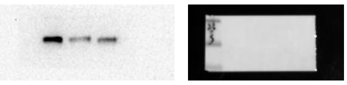

a-Tubulin: 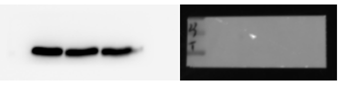

Supplement: Supplementary file 2 — Original Data File [file 41419_2022_5135_MOESM2_ESM.pdf]
