# Supplementary material for: NXN suppresses metastasis of hepatocellular carcinoma by promoting degradation of Snail through binding to DUB3
Source: Cell Death Dis. 2022 Aug 4;13(8):676. doi: 10.1038/s41419-022-05135-7 (PMC9352874; doi:10.1038/s41419-022-05135-7)
Supplement: Supplementary file 3 — supplementary figure legends and tables [file 41419_2022_5135_MOESM3_ESM.docx]

**Supplementary figure legends**

**Figure S1. NXN mRNA expression in human tumor tissues and normal tissues in TCGA database.**

NXN mRNA expression in HCC from TCGA database.

**Figure S2. Overexpression of NXN inhibited proliferation of HCC cells *in vitro*.**

(A) NXN mRNA and protein expression in immortalized human liver cell line and HCC cell lines. (B) NXN suppressed the cell growth of HCC cells, measured by CCK8 assays. Statistical results were presented as mean±SD (from triplicates), and significance was determined by Student’s t-test (**P* < 0.05).

**Figure S3. Overexpression of NXN inhibited proliferation and metastasis of HCC cells *in vivo*.**

(A) Knockdown of NXN enhanced proliferation of HCC cells in nude mice. Tumors after subcutaneous injection of indicated Hepa1-6 cells. Four mice per group. (B) Knockdown of NXN enhanced metastasis of HCC cells in nude mice. Gross images of metastatic lung tumors after tail vein injection of indicated Hepa1-6 cells. Five mice per group.

**Figure S4. The promoting effect of NXN depletion on HCC cells metastasis was rescued by the knock-down of Snail.**

(A) The efficiency of Snail overexpression and knockdown were measured by Western blot assays. (B) Knockdown of Snail attenuated NXN depletion induced increase of HCC cell metastasis in nude mice. Gross images of metastatic lung tumors after tail vein injection of indicated Hepa1-6 cells. Scale bar, 100 µm. Five mice per group. (C) The expression of downstream target genes of Snail detected by quantitative real-time PCR. The expression of NXN affects downstream target genes of Snail indicating the effects of NXN on the suppression of HCC were Snail-dependent. Statistical results were presented as mean±SD (from triplicates), and significance was determined by Student’s t-test (**P* < 0.05).

**Supplementary tables**

**Table S1 The key to all TCGA abbreviations.**

| Full name | Abbreviation |
| --- | --- |
| Cholangiocarcinoma | CHOL |
| Colon adenocarcinoma | COAD |
| Esophageal carcinoma | ESCA |
| Glioblastoma multiforme | GBM |
| Head and neck squamous cell carcinoma | HNSC |
| Kidney chromophobe | KICH |
| Kidney renal clear cell carcinoma | KIRC |
| Kidney renal papillary cell carcinoma | KIRP |
| Liver hepatocellular carcinoma | LIHC |
| Lung adenocarcinoma | LUAD |
| Lung squamous cell carcinoma | LUSC |
| Pancreatic adenocarcinoma | PAAD |
| Prostate adenocarcinoma | PRAD |
| Pheochromocytoma and Paraganglioma | PCPG |
| Rectum adenocarcinoma | READ |
| Sarcoma | SARC |
| Skin cutaneous melanoma | SKCM |
| Thyroid carcinoma | THCA |
| Thymoma | THYM |
| Stomach adenocarcinoma | STAD |
| Uterine corpus endometrial carcinoma | UCEC |

**Table S2** Correlation of clinicopathological parameters and NXN expression in TCGA cohort

| Characteristics | Low NXN (n = 103) | High NXN (n = 261) | *P* value |
| --- | --- | --- | --- |
| Age (year) | 64 (56, 70) | 59 (51, 68) | 0.057 |
| Gender (male/female) | 67/36 (65/36) | 179/82 (68.6/31.4) | 0.516 |
| BMI (>25/≤25) | 44/51 (46.3/53.7) | 112/124 (47.5/52.4) | 0.851 |
| ALB (≤35/>35) (g/L) | 56/30 (65.1/34.9) | 139/65 (68.1/31.9) | 0.617 |
| TBIL (>17.1/≤17.1) (μmol/L) | 6/82 (6.8/93.2) | 18/193 (8.5/91.5) | 0.619 |
| AFP (>400/≤400) (ng/ml) | 23/56 (29.1/70.9) | 39/157 (19.9/80.1) | 0.098 |
| Vascular invasion (yes/no) | 39/49 (44.3/55.7) | 67/156 (30.0/70.0) | 0.017* |
| Histologic grade (G1-2/ G3-4) | 40/63 (38.8/61.2) | 90/171 (34.5/65.5) | 0.435 |
| [Cirrhosis](javascript:void(0);) (yes/no) | 33/70 (32/68) | 102/159 (39.1/60.9) | 0.210 |
| TNM stage (III-IV/ I-II) | 25/74 (25.3/74.7) | 61/180 (25.3/74.7) | 0.991 |

BMI, body mass index; ALB, albumin; TBIL, total bilirubin; AFP, alpha-fetoprotein; TNM stage, tumor-node-metastasis stage; * *P* < 0.05.

**Table S3** Univariate and multivariate Cox regression analyses for overall survival in TCGA cohort

| Characteristics | Univariate analysis | | | | Multivariate analysis | | |
| --- | --- | --- | --- | --- | --- | --- | --- |
|  | HR | 95% CI | *P* value | HR | | 95% CI | *P* value |
| NXN (low/high) | 1.489 | 1.023-2.167 | 0.038* | 1.576 | | 1.067-2.329 | 0.022* |
| Age (year) (≤60/>60) | 1.188 | 0.836-1.688 | 0.336 |  | |  |  |
| Gender (male/female) | 0.861 | 0.603-1.231 | 0.412 |  | |  |  |
| BMI (>25/≤25) | 0.838 | 0.577-1.216 | 0.352 |  | |  |  |
| ALB (g/L) (≤35/>35) | 1.698 | 0.997-2.893 | 0.051 |  | |  |  |
| TBIL (μmol/L) (>17.1/≤17.1) | 1.063 | 0.463-2.439 | 0.886 |  | |  |  |
| AFP (ng/ml) (>400/≤400) | 1.112 | 0.681-1.814 | 0.672 |  | |  |  |
| Vascular invasion (yes/no) | 1.351 | 0.892-2.047 | 0.155 |  | |  |  |
| Histologic grade (G3-4/G1-2) | 1.075 | 0.752-1.537 | 0.690 |  | |  |  |
| [Cirrhosis](javascript:void(0);) (yes/no) | 0.535 | 0.361-0.792 | 0.002* | 0.660 | | 0.435-1.000 | 0.050 |
| TNM stage (III-IV/I-II) | 2.608 | 1.797-3.785 | <0.001* | 2.426 | | 1.663-3.541 | <0.001* |

HR, hazard ratio; CI, confidence interval; BMI, body mass index; ALB, albumin; TBIL, total bilirubin; AFP, alpha-fetoprotein; TNM stage, tumor-node-metastasis stage; * *P* < 0.05.
