# Supplementary figures and images for: NXN suppresses metastasis of hepatocellular carcinoma by promoting degradation of Snail through binding to DUB3
Source: Cell Death Dis. 2022 Aug 4;13(8):676. doi: 10.1038/s41419-022-05135-7 (PMC9352874; doi:10.1038/s41419-022-05135-7)

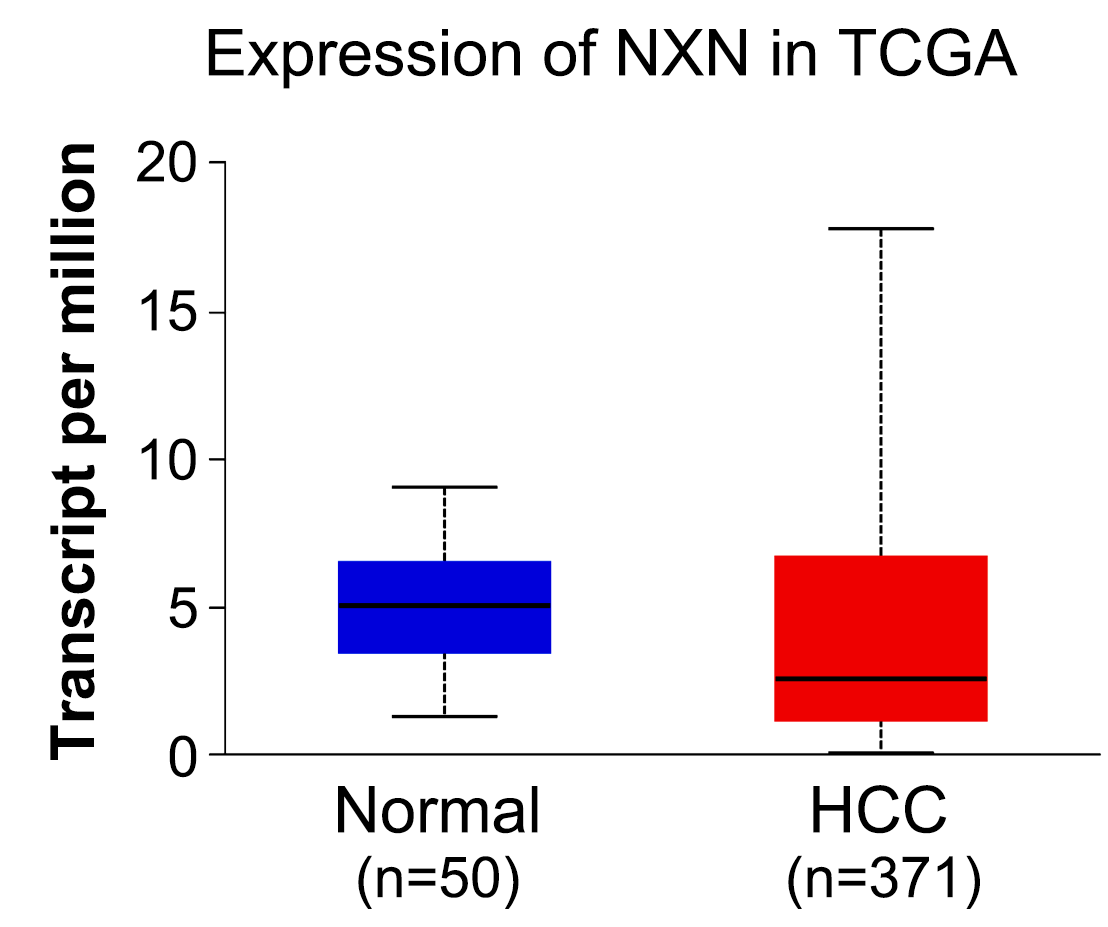

Supplement: Supplementary file 4 — Figure S1. NXN mRNA expression in human tumor tissues and normal tissues in TCGA database. [file 41419_2022_5135_MOESM4_ESM.tif]

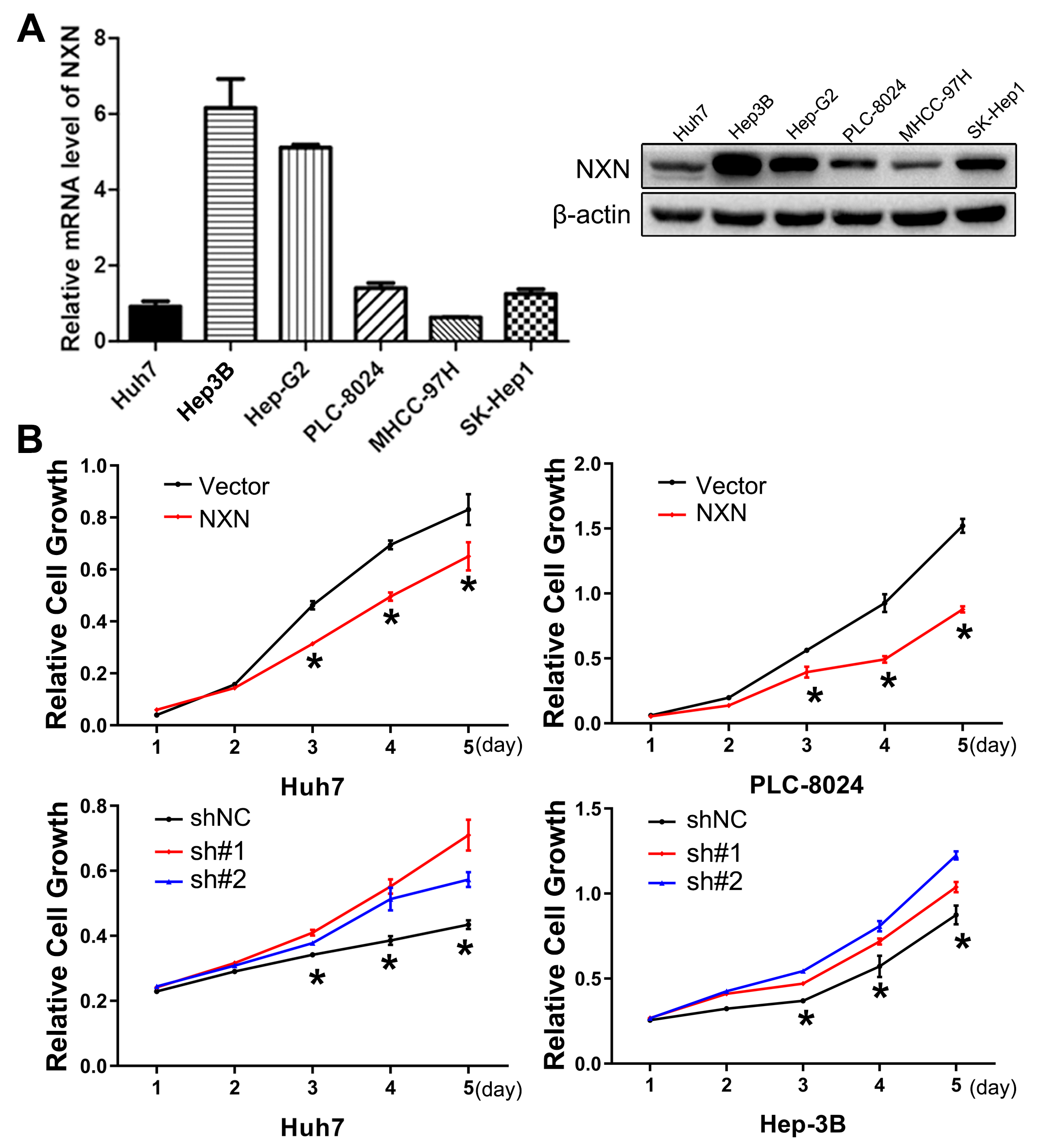

Supplement: Supplementary file 5 — Figure S2. Overexpression of NXN inhibited proliferation of HCC cells in vitro. [file 41419_2022_5135_MOESM5_ESM.tif]

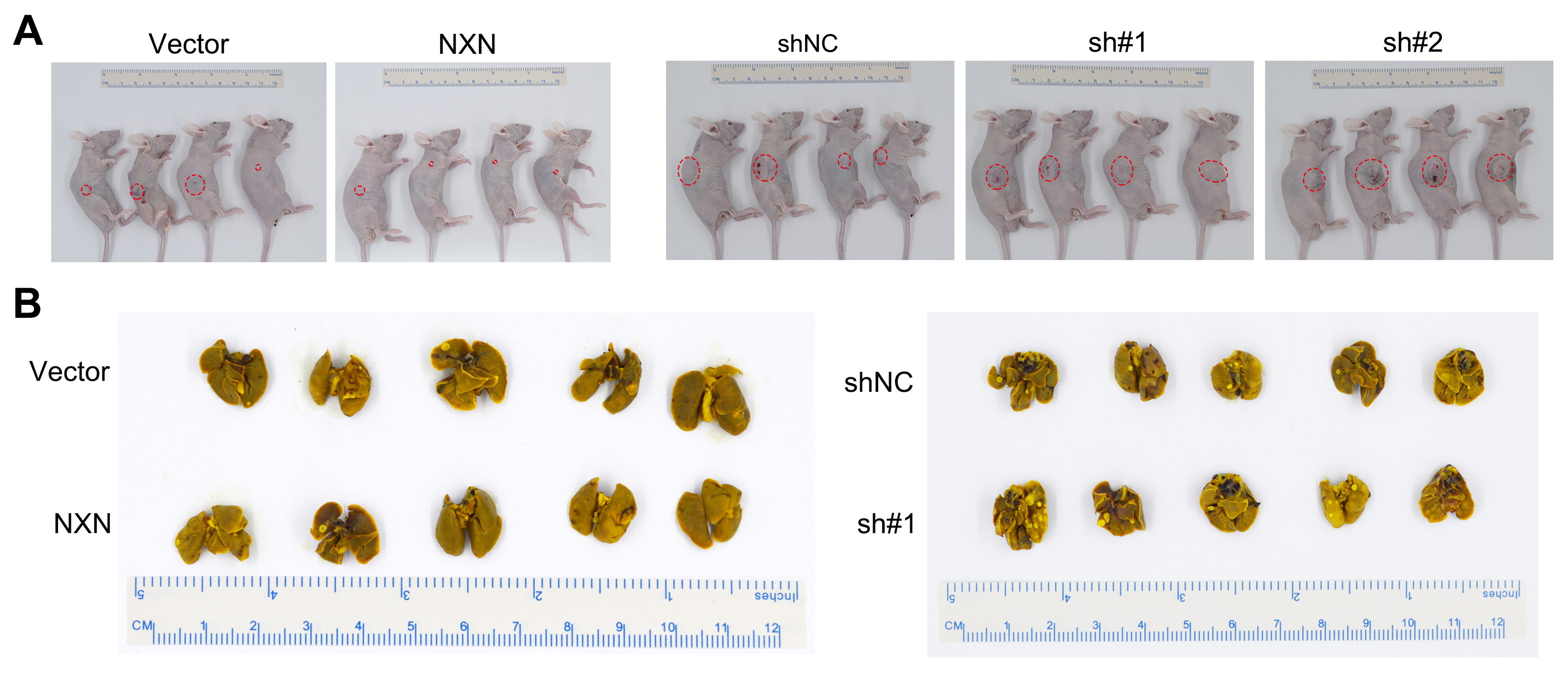

Supplement: Supplementary file 6 — Figure S3. Overexpression of NXN inhibited proliferation and metastasis of HCC cells in vivo. [file 41419_2022_5135_MOESM6_ESM.tif]

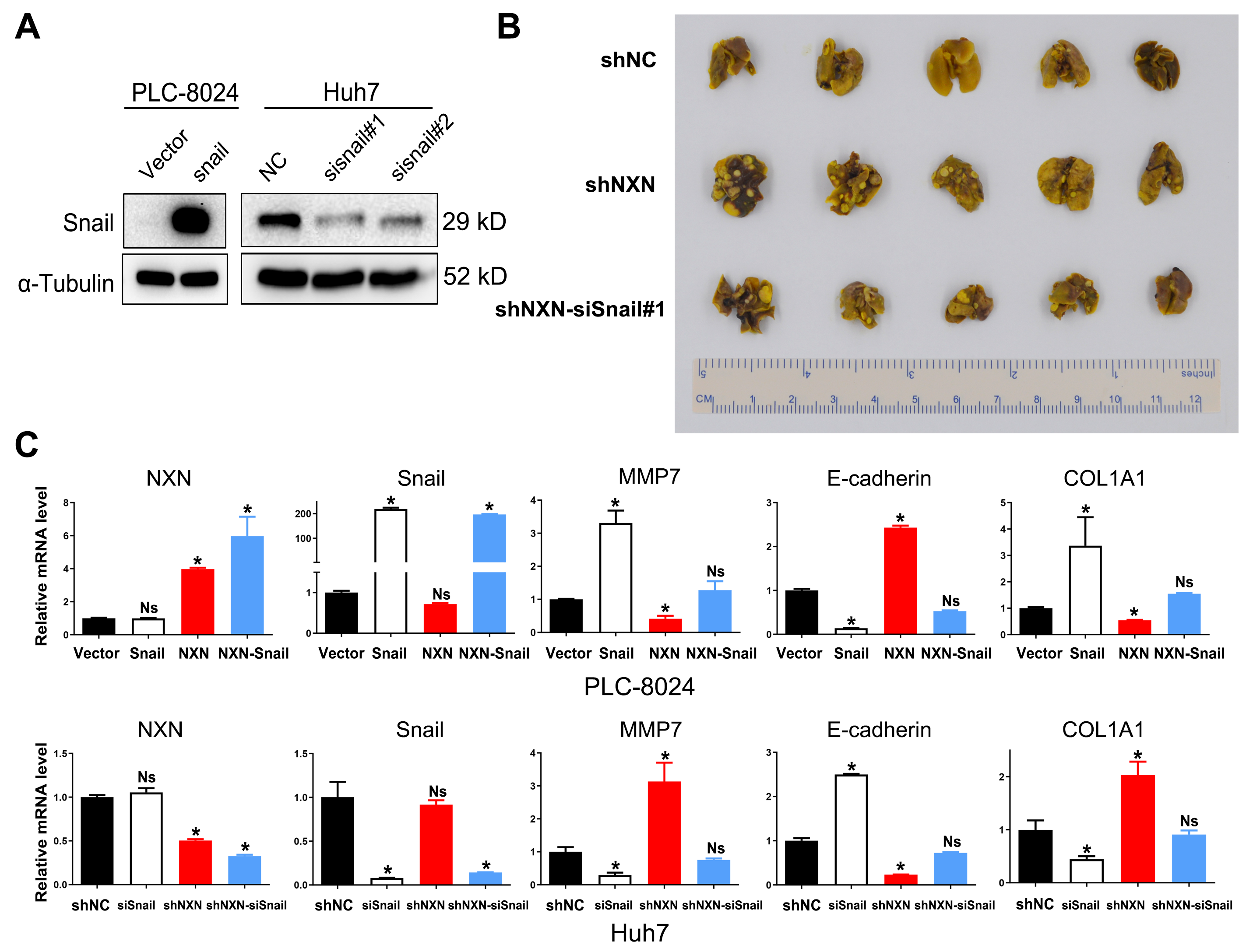

Supplement: Supplementary file 7 — Figure S4. The promoting effect of NXN depletion on HCC cells metastasis was rescued by the knock-down of Snail. [file 41419_2022_5135_MOESM7_ESM.tif]
